# Risk factors for subjective cognitive decline: the CABLE study

**DOI:** 10.1038/s41398-021-01711-1

**Published:** 2021-11-09

**Authors:** Chen Wen, Hao Hu, Ya-Nan Ou, Yan-Lin Bi, Ya-Hui Ma, Lan Tan, Jin-Tai Yu

**Affiliations:** 1 Department of Neurology, Qingdao Municipal Hospital, Qingdao University, Qingdao, China; 2 Department of Anesthesiology, Qingdao Municipal Hospital, Qingdao University, Qingdao, China; 3 Department of Neurology and Institute of Neurology, Huashan Hospital, State Key Laboratory of Medical Neurobiology and MOE Frontiers Center for Brain Science, Shanghai Medical College, Fudan University, Shanghai, China

**Keywords:** Pathogenesis, Predictive markers

## Abstract

Increasing evidences supported that subjective cognitive decline (SCD) might be a potential first symptomatic manifestation of Alzheimer’s disease (AD). The rapidly growing number of SCD individuals who seek medical help and advice also makes it urgent to develop more precise strategy for SCD. Therefore, this study aimed to explore the risk factors for SCD. Logistics and linear regression models were performed to investigate 41 factors for SCD in 1165 participants without objective cognitive impairment. Cochran-Armitage trend test was used to confirm the constant trend toward higher prevalence of SCD with an increasing number of risk factors. A high overall prevalence of SCD was found in total participants (42%). Eight factors were eventually identified as risk factors for SCD, including four stable factors associated with both SCD statues and severity (older age, thyroid diseases, minimal anxiety symptoms, and day time dysfunction; odds ratio (OR) ranging from 1.74 to 2.29) as well as four suggestive factors associated with either SCD statues or severity (female sex, anemia, lack of physical exercises, and living alone; OR ranging from 1.30 to 2.29). The prevalence of SCD gradually increased with the number of risk factors clustering increased in individuals (*p* for trend <0.001). Five of these eight factors were further proved among individuals with SCD-plus features. These findings revealed several risk factors for SCD, providing some new clues for formulating priority strategies for early prevention of SCD.

## Introduction

As the most common form of dementia, Alzheimer’s disease (AD) has become a priority worldwide in terms of both public health and social care [1]. It has been revealed that AD-related pathophysiologies begin a decade or more before the onset of objective cognitive impairment that can be measured with standardized neuropsychological scales [1]. The failure of several previous clinical trials of therapies in the dementia or mild cognitive impairment (MCI) stages further encouraged researchers to shift their focus to the preclinical stage of AD [2, 3]. Subjective cognitive decline (SCD), a cognitive state between objective cognitive impairment and intact cognition, is receiving increasing attention as the potentially first symptomatic manifestation of AD [4]. Longitudinal studies have shown that SCD participants have a higher conversion rate and shorter conversion time to MCI and dementia than cognitively intact individuals [5, 6]. Furthermore, abnormal levels of AD-related biomarkers in cerebrospinal fluid [7], increased amyloid deposition in brain measured by positron-emission tomography (PET) [8, 9] and severer brain atrophy measured by magnetic resonance imaging (MRI) [10] were also found in SCD individuals. All the above evidence confirmed that the exploration of SCD might provide important clues for a preclinical stage closely related to dementia or AD.

It has been widely accepted that genetic and environmental risk factors work together to influence the occurrence and progression of dementia. Our previous meta-analysis showed that one third of the risk factors of AD were modifiable [11], which highlighted the feasibility and importance of early prevention. However, up to now, almost all the previous studies focused on risk factors for objective cognitive impairment [11, 12], and the risk factors for SCD still remained unclear. Since the number of SCD individuals who seek medical help and advice is rapidly growing, it is necessary to detect the risk factors for SCD. In addition, although the outstanding relevance of classical risk factors for dementia was beyond debate, these factors may not be given similar priority in SCD. Therefore, our study was designed to explore risk factors for SCD in a large sample of 1165 cognitively normal (CN) Northern Han Chinese, aiming to provide new clues to early prevention and intervention of SCD.

## Methods

### Participants

All analyses were performed on the data from the Chinese Alzheimer’s Biomarker and LifestylE (CABLE) study. Initiated in 2017, CABLE study is an ongoing large-scale cohort study majorly focused on AD risk factors and biomarkers in the northern Chinese Han population [13]. The exclusion criteria include: (1) central nervous system infection, head trauma, multiple sclerosis, or other major neurological disorders; (2) major psychological disorders; (3) severe systemic diseases that may affect CSF or blood levels of AD biomarkers including Aβ and tau; and (4) family history of genetic diseases. All participants underwent comprehensive clinical, neuropsychological, psychosocial, and psychiatric evaluations to determine their cognitive diagnoses in compliance with the National Institute on Aging–Alzheimer’s Association (NIA-AA) workgroup diagnostic criteria [14, 15]. The objective cognition was tested by Chinese-modified mini-mental state examination (CM-MMSE: ≤24 for >6 years of education, ≤20 for 1~6 years of education, ≤17 for 0 year of education) and Montreal Cognitive Assessment (MOCA: <24 for >12 years of education, <22 for 7~12 years of education, <19 for <7 years of education). The subjective cognition was tested by a subjective cognitive decline (SCD) scale (detailed below).

### Standard protocol approvals, registrations, and patient consents

The CABLE study gained the approval of institutional review board of Qingdao Municipal Hospital. The study procedure was conducted strictly in accordance with the principles of the Declaration of Helsinki and written informed consent was obtained from all participants or their guardians.

### Basic information

Basic information of participants were collected including age (<65 years or ≥65 years), sex (male or female), years of education (continuous), and lifestyle factors, including living alone, habit of drinking coffee, habit of drinking tea, lack of physical exercises, living in urban areas, smoking status, and alcohol status were collected through a dichotomy questionnaire (yes or no). Participants’ medical history (yes or no) and current medication information (yes or no) were also collected, including stroke, hypertension, diabetes mellitus, coronary disease, hyperlipoidemia, kidney diseases, cancer, anemia, thyroid diseases, use of anti-hypertension drugs, use of anti-diabetes drugs, and use of vitamins. All the information would be confirmed by available clinical information in the electronic medical record system in Qingdao Municipal Hospital.

### Assessment of SCD

The questionnaire of SCD was based on SCD-I recommendations [4, 16]. Two assessment methods, classification and continuous indicators, were used to identify the subjective memory function. People were thought to have SCD status if they answered “yes” for the question “Do you think your memory is declining compared to what it used to be?”, which could not be explained by other diseases or drug abuse. A continuous SCD scale was used to reflect the severity of SCD (see e-Method). Adopting the form of Likert scale and combining with Top nine SCD items [17], it was adapted from subjective memory decline scale [18]. After the adaptation, a subject can score 0–2 points for each question and the greatest total score for 6 questions in the questionnaire is 12 points. Participants would get higher score if they had more serious SCD. At the same time, we also collected the onset time of SCD status, whether the SCD status was confirmed by an observer and whether there were subjective impairments in cognitive domains other than memory (such as difficulty with language or finding words, decreased ability of organization, decreased ability of decision-making and decreased attention).

Despite the growing interest in SCD as the putative first syndrome stage of AD, some evidence also indicated that non-AD medical problems could also underlie SCD. To select SCD individuals who had particularly high risk of objective cognitive decline and an increased likelihood for preclinical AD, a list of SCD-features (SCD-plus) was recommended [19]. Based on this recommendation, 139 participants who met at least three features were classified into a SCD-plus subgroup.

### Neuropsychiatric scales and PSQI

Neuropsychiatric symptoms were tested by Hamilton anxiety scale (HAMA) and Hamilton depression scale (HAMD). Participants included in our study did not have significant anxiety (HAMA > 7) and depression (HAMD > 7). Minimal anxiety symptoms (MAS) were defined as 1≤ HAMA score <7, and minimal depression symptoms (MDS) were defined as 1≤ HAMD score <7 [20].

PSQI scale included sleep quality (bad or good), sleep latency (minutes taken from going to bed to falling asleep), sleep duration (hours), bedtime (the usual time to go to bed), sleep efficiency (the ratio of sleep duration-to-time spent in bed), sleep disorders (abnormal behaviors during sleep), sleep assistance (medication from doctors’ prescription or pharmacy to aid sleep), and day time dysfunction (the phenomenon that individuals who are too sleepy to finish daily activities during the day time). All of the above scales were evaluated by professional neurological physicians. In this study, a subset (*n* = 647, CN = 347, SCD = 300) with complete neuropsychiatric scales and PSQI was used to test these factors.

### *APOE* gene and laboratory indicators of blood

The blood samples were stored in enzyme-free EP tube at –80 °C before DNA was extracted. The *APOE ε4* carrier was defined as the carrier of rs7412 or rs429358 with the assistance of restriction fragment length polymorphism (RFLP) technology using QIAamp^®^ DNA Blood Mini Kit (250).

The laboratory blood samples were collected into a blood tube containing silica by vein puncture after participants had been fasting for at least 8 h. Blood samples were tested at Clinical Chemistry Laboratory at Qingdao Municipal Hospital. The samples were centrifuged at 3000*g* for 10 min to obtain serum. Fasting blood glucose (FBG) levels were measured by glucose hexokinase (HK) method using Glucose Reagent (Ningbo Ruiyuan Biotechnology Co., Ltd, China). Blood urea nitrogen (BUN) levels were measured by urease glutamic acid dehydrogenase (UV liquid) method using Urea Test Kit (Ningbo Ruiyuan Biotechnology Co., Ltd, China). Creatinine (CR) levels were measured by sarcosine oxidase method using Creatinine Test Kit (Ningbo Ruiyuan Biotechnology Co., Ltd, China). Uric acid (UA) levels were determined by uricase method using Uric Acid Test Kit (Ningbo Ruiyuan Biotechnology Co., Ltd, China). Triglyceride (TG) levels were measured by glycerol phosphorus oxidase peroxidase (GPO-PAP) method using Triglycerides Test Kit (Ningbo Ruiyuan Biotechnology Co., Ltd, China). Total cholesterol (TC) levels were measured by cholesterol esterase peroxidase (CHOD-PAP) method using Cholesterol total Test Kit (Ningbo Ruiyuan Biotechnology Co., Ltd, China). Low-density lipoprotein cholesterol (LDL-C) and high-density lipoprotein cholesterol (HDL-C) levels were measured by homogeneous method using Creatinine Test Kit (Beckman Coulter Biotechnology (Suzhou) Co., Ltd, China).

### Statistical analysis

To describe the variables, we calculated mean ± SD for continuous variables and number (prevalence) for categorical variables. Differences between the two groups were analyzed by Chi-square tests for categorical variables and Wilcoxon tests for numerical variables. False discovery rate (*q* value) was used to adjust for multiple comparisons.

Risk factors were determined using three models. Firstly, univariate logistic regression models (Model 1) were used to estimate the odds ratio (OR) and 95% confidence interval (CI) for the association of each factor with the risk of SCD. Then all the significant factors in univariate models (*p* < 0.1) were included in two multivariate models, including the multivariate logistic regression for SCD status (Model 2) and multivariate linear regression for SCD severity (Model 3). In addition, we included age, sex, years of education, and APOE *ε4* status in two multivariate models as the basic covariates, no matter whether they were significant or not in Model 1. Furthermore, we conducted subgroup analyses of these risk factors according to age (midlife <65; late life ≥65) and sex (male; female). Then, the Cochran-Armitage trend test was used to confirm the constant trend toward higher prevalence of SCD with an increasing number of risk factors. Finally, we repeated the above three analyses (Model 1–3) in a post hoc analysis to explore the risk factors for SCD-plus.

The multicollinearity was assessed using variance inflation factor (VIF). No multicollinearity existed in each model of the current study. A two-tailed *p* < 0.05 was considered significant except where specifically noted. Analyses were carried out using R-3.6.1.

## Results

### Characteristics of participants

A total of 1165 participants were included from the CABLE study consisting of 672 CN controls and 493 SCD participants (Table 1). All participants were cognitively unimpaired (mean CM-MMSE score = 27.95). Female participants accounted for 58.6% and *APOE ε4* carriers accounted for 15.45%. Compared with CN individuals, SCD participants were older, more likely to be living alone, having greater percent of hypertension, diabetes mellitus and thyroid diseases, and worse sleep quality (all *p* values < 0.05).

**Table 1:** Characteristics of participants.

Variables	CN (672)	SCD (493)	Total (1165)	*p*	*q*
Age (≥65 years)	235 (34.97%)	248 (50.30%)	483 (41.46%)	<0.01	<0.01
Sex (female)	254 (37.80%)	227 (46.04%)	481 (41.29%)	0.01	0.02
Education (years)	10.07 ± 4.31	9.81 ± 4.51	9.96 ± 4.40	0.88	0.88
CM-MMSE score	28.07 ± 1.99	27.78 ± 2.21	27.95 ± 2.09	0.28	0.37
*APOE ε*4 carrier	100 (14.88%)	80 (16.23%)	180 (15.45%)	0.59	0.66
SCD severity scale	0.15 ± 0.75	2.28 ± 2.14	1.30 ± 1.96	<0.01	<0.01
Lifestyle					
Smoking (yes)	212 (31.55%)	142 (28.80%)	354 (30.39%)	0.31	0.39
Alcohol (yes)	215 (31.99%)	137 (27.79%)	352 (30.21%)	0.14	0.23
Living alone (yes)	22 (3.27%)	41 (8.32%)	63 (5.41%)	<0.01	<0.01
Coffee (yes)	81 (12.05%)	56 (11.36%)	137 (11.76%)	0.79	0.82
Tea (yes)	421 (62.65%)	322 (65.31%)	743 (63.78%)	0.38	0.46
Lack physical exercises (yes)	341 (50.74%)	294 (59.63%)	635 (54.51%)	<0.01	0.01
Living in urban (yes)	492 (73.21%)	380 (77.08%)	872 (74.85%)	0.15	0.24
Clinical diseases					
Stroke (yes)	14 (2.08%)	22 (4.46%)	36 (3.09%)	0.03	0.07
Hypertension (yes)	227 (33.78%)	212 (43.00%)	439 (37.68%)	<0.01	0.01
Diabetes mellitus (yes)	82 (12.20%)	92 (18.66%)	174 (14.94%)	<0.01	0.01
Coronary disease (yes)	76 (11.31%)	82 (16.63%)	158 (13.56%)	0.01	0.04
Hyperlipoidemia (yes)	21 (3.13%)	23 (4.67%)	44 (3.78%)	0.23	0.33
Kidney diseases (yes)	19 (2.83%)	21 (4.26%)	40 (3.43%)	0.24	0.34
Cancer (yes)	38 (5.65%)	31 (6.29%)	69 (5.92%)	0.74	0.81
Anemia (yes)	33 (4.91%)	40 (8.11%)	73 (6.27%)	0.04	0.07
Thyroid diseases (yes)	56 (8.33%)	78 (15.82%)	134 (11.5%)	<0.01	0.00
Anti-hypertension drug (yes)	156 (23.21%)	142 (28.80%)	298 (25.58%)	0.04	0.07
Anti- diabetes drug (yes)	60 (8.93%)	67 (13.59%)	127 (10.90%)	0.02	0.04
Vitamins (yes)	65 (9.67%)	72 (14.60%)	137 (11.76%)	0.01	0.04
Scale*					
HAMA score (MAS)	48 (13.79%)	83 (27.57%)	131 (20.18%)	<0.01	<0.01
HAMD score (MDS)	49 (14.08%)	74 (24.58%)	123 (18.95%)	<0.01	<0.01
PSQI					
Sleep quality (bad)	63 (9.38%)	89 (18.05%)	152 (13.05%)	<0.01	<0.01
Sleep latency	22.55 ± 25.29	29.19 ± 31.53	25.63 ± 28.53	<0.01	0.01
Sleep duration (hours)				0.04	0.06
≤5	57 (16.38%)	67 (22.26%)	124 (19.11%)		
5–6	61 (17.53%)	68 (22.59%)	129 (19.88%)		
6−7	95 (27.30%)	81 (26.91%)	176 (27.12%)		
7–8	100 (28.74%)	64 (21.26%)	164 (25.27%)		
>8	35 (10.06%)	21 (6.98%)	56 (8.63%)		
Bedtime				0.12	0.13
Before 8:00 p.m.	38 (10.92%)	27 (8.97%)	65 (10.02%)		
8:00–9:00 p.m.	81 (23.28%)	54 (17.94%)	135 (20.80%)		
9:00–10:00 p.m.	142 (40.80%)	124 (41.20%)	266 (40.99%)		
10:00–11:00 p.m.	74 (21.26%)	73 (24.25%)	147 (22.65%)		
After 11:00 p.m.	13 (3.74%)	23 (7.64%)	36 (5.55%)		
Sleep efficiency (≤70%)	57 (16.38%)	61 (20.27%)	118 (18.18%)	0.28	0.28
Sleep disorders	248 (71.26%)	235 (78.07%)	483 (74.42%)	0.05	0.06
Sleep assistance	20 (5.75%)	37 (12.29%)	57 (8.78%)	0.01	0.01
Day time dysfunction	16 (4.60%)	31 (10.30%)	47 (7.24%)	0.01	0.01
Laboratory indicators					
FBG (mmol/L)	5.53 ± 1.14	5.60 ± 1.06	5.56 ± 1.11	0.07	0.18
BUN (mmol/L)	5.76 ± 1.49	5.92 ± 1.41	5.83 ± 1.46	0.10	0.21
CR (μmol/L)	67.88 ± 14.52	68.79 ± 14.95	68.27 ± 14.70	0.39	0.62
UA (μmol/L)	360.34 ± 86.57	360.45 ± 83.37	360.38 ± 85.17	0.88	0.88
TG (mmol/L)	1.53 ± 1.26	1.42 ± 0.80	1.48 ± 1.09	0.59	0.67
TC (mmol/L)	4.83 ± 0.98	4.95 ± 1.02	4.88 ± 1.00	0.04	0.15
HDL-C (mmol/L)	1.20 ± 0.28	1.20 ± 0.26	1.20 ± 0.27	0.58	0.67
LDL-C (mmol/L)	2.83 ± 0.68	2.91 ± 0.72	2.86 ± 0.70	0.04	0.15

Continuous variables are presented as mean ± SD and categorical variables as number (percentage).

Abbreviations: *CN* cognitive normal, *SCD* subjective cognitive decline, *MMSE* mini-mental state examination, *APOE ε4* apolipoprotein E ε4, *FBG* fasting blood glucose, *BUN* blood urea nitrogen, *CR* creatinine, *UA* uric acid, *TG* triglyceride, *TC* total cholesterol, *LDL-C* low-density lipoprotein cholesterol, *HDL-C* high-density lipoprotein cholesterol, *HAMA* Hamilton anxiety scale, *MAS* minimal anxiety symptoms, *HAMD* Hamilton depression scale, *MDS* minimal depression symptoms, *PSQI* Pittsburgh sleep quality index.

Differences between two groups were analyzed by Chi-square tests for categorical variables and Wilcoxon tests for numerical variables.

*q*: Significance after false discovery rate (FDR) correction.

*A subset (*n* = 647, CN = 347, SCD = 300) with complete neuropsychiatric scales and PSQI.

### Factors associated with SCD

**Fig. 1:**
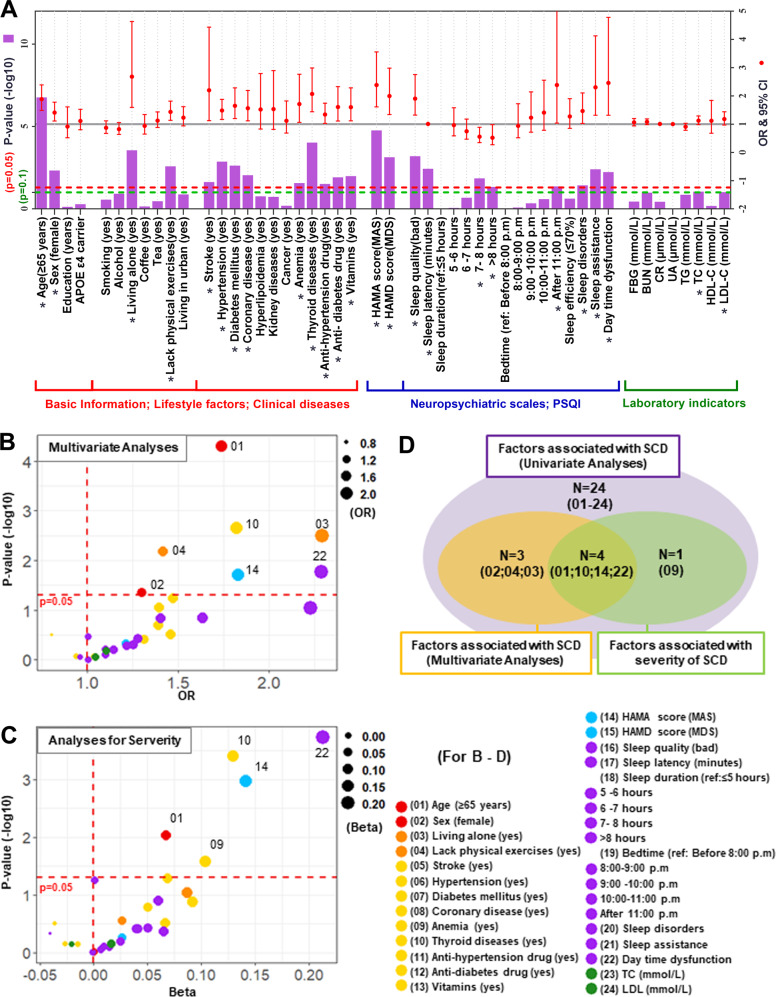
Risk factors for SCD. Risk factors for SCD were determined using three models. **A** Univariate logistic regression models (Model 1) were used to test association of each factor with the risk of SCD. **B** Then all the significant factors in univariate models (^*^p < 0.1) were included in the multivariate logistic regression (Model 2) to test their associations with the SCD status. **C** Similarly, all the significant factors in univariate models (^*^p < 0.1) were also included in the multivariate linear regression (Model 3) to test their associations with the SCD severity. The age, sex, years of education, and *APOE ε4* status were included in two multivariate models as the basic covariates, regardless of their results in Model 1. **D** The significant results of three models were summarized in a Venn diagram. Abbreviations: SCD: subjective cognitive decline; OR: odds ratio; LCI: lower confidence interval (2.5%); UCI: upper confidence interval (97.5%); APOE ε4: apolipoprotein E ε4; FBG: fasting blood glucose; BUN: blood urea nitrogen; CR: creatinine; UA: uric acid; TG: triglyceride; TC: total cholesterol; LDL-C: low-density lipoprotein cholesterol; HDL-C: high-density lipoprotein cholesterol; HAMA: Hamilton anxiety scale; MAS: minimal anxiety symptoms; HAMD: Hamilton depression scale; MDS: minimal depression symptoms; PSQI: Pittsburgh sleep quality index.

Firstly, 24 factors were screened out in univariate analyses (Model 1), including older age (≥65 years: OR 1.88, 95% CI 1.49–2.39), female sex (OR 1.40, 95% CI 1.11–1.78), living alone (OR 2.68, 95% CI 1.59–4.63), lack of physical exercises (OR 1.43, 95% CI 1.13–1.82), eight disease-related factors (stroke: OR 2.20, 95% CI 1.12–4.43; hypertension: OR 1.48, 95% CI 1.16–1.88; diabetes mellitus: OR 1.65, 95% CI 1.19–2.28; coronary disease: OR 1.56, 95% CI 1.12–2.19; anemia: OR 1.71, 95% CI 1.06–2.77; thyroid disease: OR 2.07, 95% CI 1.44–2.99; anti-hypertension drugs: OR 1.34, 95% CI 1.03–1.74; anti-diabetes drugs: OR 1.60, 95% CI 1.11–2.33; vitamins: OR 1.60, 95% CI 1.12–2.29), MAS (OR 2.38, 95% CI 1.61–3.56), MDS (OR 1.99, 95% CI 1.34–2.98), seven sleep-related factors (bad sleep quality: OR 1.90, 95% CI 1.32–2.76; longer sleep latency: OR 1.01, 95% CI 1.00–1.02; sleep duration (reference: ≤5 h; 7–8 h: OR 0.55, 95% CI 0.34–0.89; >8 h: OR 0.52, 95% CI 0.27–0.98); bed time (reference: before 8:00 p.m.; after 11:00 p.m.: OR 2.38, 95% CI 1.04–5.66); sleep disorders: OR 1.46, 95% CI 1.02–2.10; sleep assistance: OR 2.30, 95% CI 1.32–4.78; day time dysfunction: OR 2.46, 95% CI 1.32–4.78), and two laboratory indicators of peripheral blood (TC: OR 1.13, 95% CI 0.98–1.29; LDL-C: OR 1.18, 95% CI 0.97–1.44) (Fig. 1A and Table S1).

Then, seven of these 24 factors still had significant associations with SCD risk in multivariate logistic regression models (Model 2), including older age (≥65 years: OR 1.74, 95% CI 1.33–2.27), female sex (OR 1.30, 95% CI 1.01–1.69), living alone (OR 2.29, 95% CI 1.33–4.04), lack of physical exercises (OR 1.42, 95% CI 1.10–1.82), thyroid disease (OR 1.82, 95% CI 1.24–2.69), MAS (OR 1.83, 95% CI 1.10–3.06), and day time dysfunction (OR 2.29, 95% CI 1.17–4.63) (Fig. 1B and Table S2).

Finally, five of these 24 risk factors were further proved associated with SCD severity in multivariate linear regression models (Model 3), including older age (≥65 years: Beta 0.07, 95% CI 0.02–0.12), anemia (Beta 0.10, 95% CI 0.01–0.19), thyroid disease (Beta 0.13, 95% CI 0.06–0.20), MAS (Beta 0.14, 95% CI 0.06–0.23), and day time dysfunction (Beta 0.21, 95% CI 0.10–0.32) (Fig. 1C and Table S3).

Overall, as shown in Fig. 1D, a total of eight risk factors were found associated with SCD in multivariate models, including four stable factors proved by two multivariate models (Model 2 and Model 3: older age, thyroid diseases, MAS, and day time dysfunction) as well as four suggestive factors proved by one of the two multivariate models (Model 2: female sex, lack of physical exercises, and living alone; Model 3: anemia).

### Subgroup analyses by age and sex

**Fig. 2:**
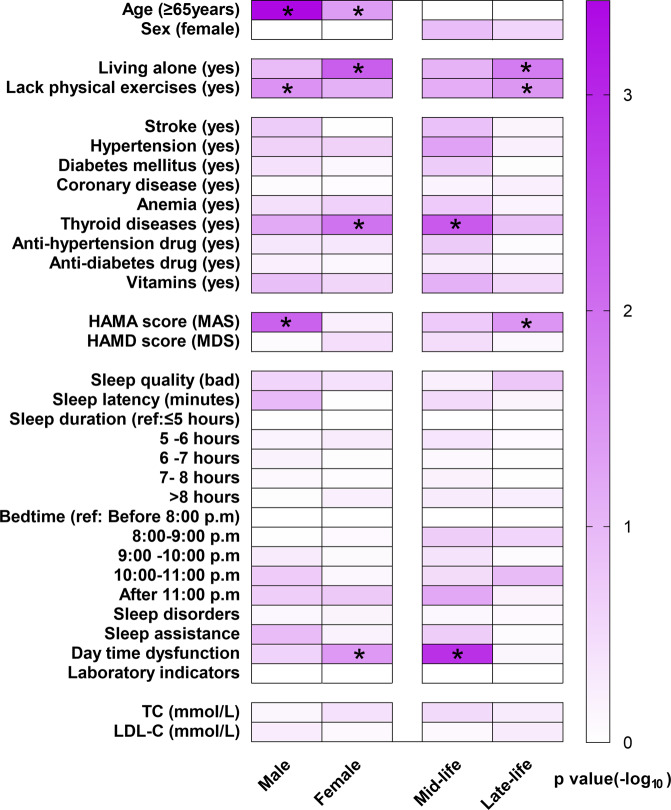
Subgroup analyses by age and sex. Multivariate logistic regression was used to test associations between factors and SCD. Abbreviations: TC: total cholesterol; LDL-C: low-density lipoprotein cholesterol; HAMA: Hamilton anxiety scale; MAS: minimal anxiety symptoms; HAMD: Hamilton depression scale; MDS: minimal depression symptoms.

Subgroup analyses showed different distribution of risk factors. Living alone, thyroid diseases, and daytime dysfunction were more likely to increase the risk of SCD in females, while lack of physical exercises and MAS increased the risk of SCD in males. Older age was the risk factor of SCD in both female and male subgroups. As for different age subgroups, living alone, lack of physical exercises, and MAS increased the risk of SCD in late life, while thyroid diseases increased the risk of SCD in midlife (Fig. 2 and Tables S4 and S5).

### Trend test and post hoc analyses

**Fig. 3:**
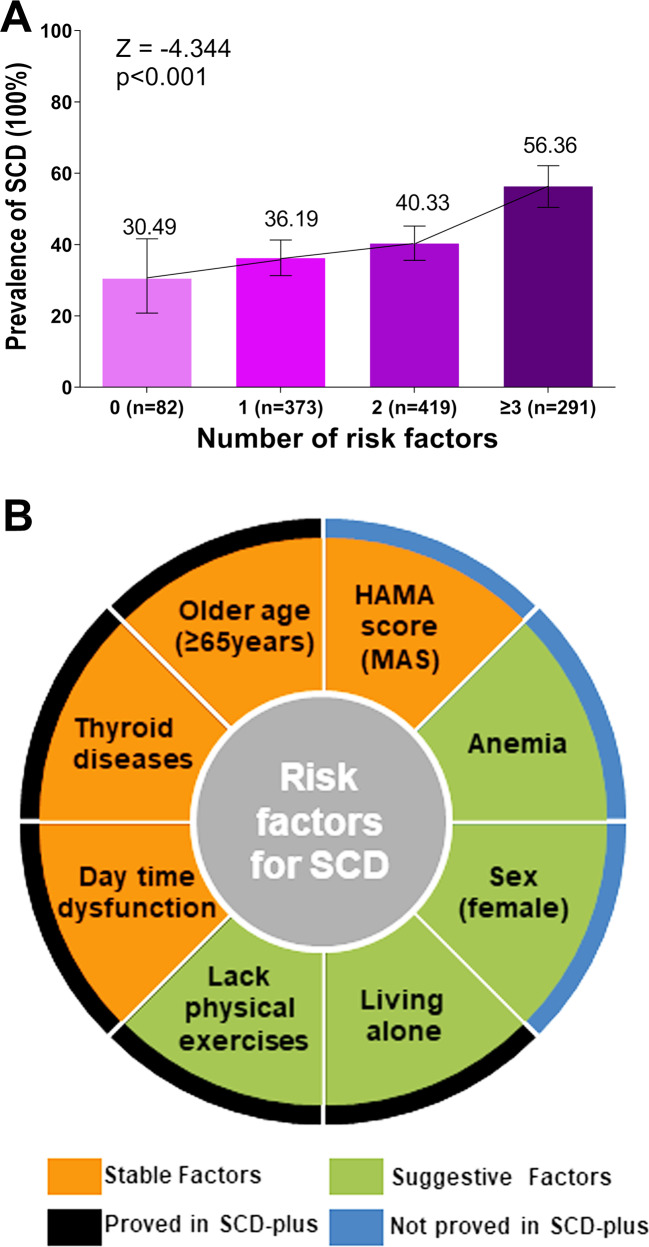
Trend test and the summary of risk factors. **A** The Cochran-Armitage trend test was used to confirm the constant trend toward higher prevalence of SCD with an increasing number of risk factors. **B** A summary chart of risk factors was established. A total of eight factors were found associated with SCD including four stable factors proved by two multivariate models and four suggestive factors proved by one of the two multivariate models. Five of the above eight factors were verified as risk factors for SCD-plus. Abbreviations: SCD: subjective cognitive decline; HAMD: Hamilton depression scale; MDS: minimal depression symptoms.

As shown in Fig. 3A, the risk of SCD gradually increased with the number of risk factors clustering in single individuals (*p* for trend <0.001). Furthermore, five of the eight risk factors for SCD were proved in the post hoc analyses between CN and SCD-plus, including older age, thyroid diseases, day time dysfunction, lack of physical exercises, and living alone (Fig. S1).

## Discussion

This study explored the risk factors for SCD in a large cohort of participants without objective cognitive impairment. Based on this population, eight factors were eventually identified as risk factors for SCD, including four stable factors (older age, thyroid diseases, MAS, and day time dysfunction) and four suggestive factors (female sex, anemia, lack of physical exercises, and living alone) (Fig. 3B). These findings filled a gap in the field of initial cognitive symptoms and might facilitate a better understanding of the pathophysiological processes involved in the initial stage of cognitive impairment, which might provide new clues to early prevention and intervention.

Notably, we found a high overall prevalence of SCD in total participants (42%). This prevalence in late life reached 51% which was consistent with the previous results varying from 50% to 80% [19, 21, 22]. It was worth noting that though this prevalence in midlife decreased, it also reached 36%. This high prevalence further highlighted the urgency of recognizing initial symptoms of cognitive impairment and their risk factors. Overall, the risk factors for SCD identified in our study were largely supported by previous evidence on AD or dementia. Both older age and female sex are classic risk factors for dementia. Our results on SCD further suggested that the influences of these two factors on cognition already existed as early as the initial stage of symptoms.

Anemia and thyroid diseases were found to increase the risk of SCD in our study. As for anemia, a study based on two independent cohorts showed that lower hemoglobin levels in blood were associated with poor cognitive function and a subsequent Mendelian randomization analysis in the same study further proved that anemia did have a primary causal impact on cognitive impairment in AD [23]. Furthermore, neuroimaging studies also related decreased hemoglobin levels to cortical thinning, white matter hyperintensities, and low cerebral perfusion [24, 25]. As for thyroid diseases, both hyperthyroidism and hypothyroidism were found associated with cognitive impairment or AD [26]. Consistent with our results in midlife, these associations seemed to be more significant in younger adults [26, 27]. Although some other diseases, such as hypertension and diabetes mellitus, were also found associated with dementia or AD [11], our results suggested that anemia and thyroid diseases might be more likely to affect the occurrence of SCD in the early stage of the disease.

An inverse association of physically exercise with the risk of cognitive impairment was widely documented. A meta-analysis that included more than 160,000 participants showed a 45% reduction in the risk of developing AD due to regular physical exercise [28]. It was important to note that in addition to long-term exercises starting from midlife, late-onset exercise interventions in late life also showed obvious effects on delaying brain aging [29]. Furthermore, living alone is a proxy measure of social isolation. A recent meta-analysis proved that living alone was a more important risk factor for dementia than previously identified and 8.9% of the incident dementia in late life (≥65 years) was attributable to living alone [30]. Consistent with this result, our study suggested that living alone might increase the risk of SCD especially in late life. All these findings indicated some important roles of social isolation in cognitive function. However, since previous systematic reviews demonstrated that loneliness was also significantly associated with incident dementia [31], whether the relationship between living alone and SCD was mediated by loneliness still need to be further explored in future studies.

As for neuropsychiatric symptoms, we identified MAS as a stable risk factor for SCD, while MDS was only significant in univariate analyses. Numerous previous studies showed that clinically significant psychiatric symptoms, including anxiety and depression, were associated with brain aging and dementia [32, 33]. There were limited studies focused on minimal psychiatric symptoms. Our recent study showed that even minimal psychiatric symptoms might promote AD-related pathologies and increased the risk of dementia [20]. In addition, recent study focused on SCD individuals also linked psychiatric symptoms to SCD, and showed that individuals with co-occurring SCD and anxiety symptoms had a 25% probability of developing MCI or dementia by 3.1 years [34, 35]. All the above evidence suggested that individuals with psychiatric symptoms, even with minor changes in psychiatric symptoms, should be alert to the risk of cognitive impairment. In addition, accumulating evidence suggested that sleep was closely related to cognitive performance and brain health [36]. In our study, daytime dysfunction was selected from eight sleep indicators as a stable risk factor for SCD. However, the relationships of sleep with cognition and AD-related pathologies seemed to be more complex and heterogeneous across different sleep indicators, and nonlinear relationships have been found by our team and other research groups [13, 36, 37]. Even so, the identification of this risk factor for SCD suggested that sleep might affect cognition at an earlier stage than we expected.

Some strengths enhanced the reliability of our study, including large sample sizes, the use of two SCD measurements, and the adoption of the latest SCD-plus features (five of the above eight factors were verified as risk factors for SCD-plus). There were still some limitations in our study. Firstly, this was a cross-sectional study, which means that the causal relationships between these risk factors and SCD could not be established and still need to be explored in longitudinal studies. Secondly, all participants in our studies were Northern Han Chinese. Our findings should be replicated in other ethnic groups. Thirdly, though this study described a preliminary outline of risk factors for SCD and gave several important suggestions, the more detailed mechanisms of these associations should be further explored in future studies. Fourthly, since SCD may be caused by early pathologies of other types of dementia, combining AD-related biomarkers (such as Aβ or phosphorylated tau in CSF or plasma) to address whether the detected risk factors are specifically related to SCD caused by early AD pathology will be an important direction for future research.

In summary, a high overall prevalence of SCD was found among population without objective cognitive impairments. We identified older age, female sex, anemia, thyroid diseases, lack of physical exercises, living alone, MAS, and day time dysfunction as risk factors for SCD. These findings further deepened the understanding of SCD and provided some new clues for formulating priority strategies for early prevention and intervention of dementia or AD.

## Supplementary information

Supplementary file.
