# Supplementary material for: Risk factors for subjective cognitive decline: the CABLE study
Source: Transl Psychiatry. 2021 Nov 9;11:576. doi: 10.1038/s41398-021-01711-1 (PMC8578345; doi:10.1038/s41398-021-01711-1)
Supplement: Supplementary file 1 — Supplementary file. [file 41398_2021_1711_MOESM1_ESM.doc]

**List of supplementary**

**e-Method. A continuous score for the severity of SCD**

**Table S1. Univariate logistic regression analysis for risk factors (Model 1)**

**Table S2. Multivariate logistic regression analysis for risk factors (Model 2)**

**Table S3. Multivariate linear regression analysis for risk factors (Model 3)**

**Table S4. Subgroup analyses stratified by sex**

**Table S5. Subgroup analyses stratified by age**

**Figure S1. Risk factors for SCD-plus**

**e-Method. A continuous score for the severity of SCD**

**Q1: Recently, did you feel the decline in memory for recent events compared with the past?**

A. Yes, much worse than before

B. Yes, slightly worse than before

C. No, exactly the same

**Q2: Recently, did you feel that you hardly remembered where you placed the objects compared with the past?**

A. Yes, much worse than before

B. Yes, slightly worse than before

C. No, exactly the same

**Q3: Recently, did you feel that you had difficulty in remembering the conversations taking place 3-5 days ago, compared with the past?**

A. Yes, much worse than before

B. Yes, slightly worse than before

C. No, exactly the same

**Q4: Recently, did you feel that you hardly remembered the things you have already arranged before compared with the past?**

A. Yes, much worse than before

B. Yes, slightly worse than before

C. No, exactly the same

**Q5: How often did you have the following problem?--having difficulty in remembering some of your special dates, such as your birthday**

A. Always

B. Sometimes

C. Never

**Q6: How often did you have the following problem?--forgetting the frequently used phone numbers**

A. Always

B. Sometimes

C. Never

**Total score: _____points (A=2; B=1; C=0; Total score =0-12 points)**

**Table S1. Univariate logistic regression analysis for risk factors (Model 1)**

| **Factors** | **p** | **OR** | **LCI** | **UCI** |
| --- | --- | --- | --- | --- |
| **Age (≥65 years)** | <0.0001 | 1.88 | 1.49 | 2.39 |
| **Sex (female)** | 0.0048 | 1.40 | 1.11 | 1.78 |
| **Education (years)** | 0.7464 | 0.91 | 0.52 | 1.61 |
| ***APOE ε*4 carrier** | 0.5300 | 1.11 | 0.80 | 1.52 |
| **Smoking (yes)** | 0.2796 | 0.87 | 0.67 | 1.12 |
| **Alcohol (yes)** | 0.1228 | 0.82 | 0.63 | 1.05 |
| **Living alone (yes)** | 0.0003 | 2.68 | 1.59 | 4.63 |
| **Coffee (yes)** | 0.7162 | 0.94 | 0.65 | 1.34 |
| **Tea (yes)** | 0.3498 | 1.12 | 0.88 | 1.43 |
| **Lack physical exercises (yes)** | 0.0026 | 1.43 | 1.13 | 1.82 |
| **Living in urban (yes)** | 0.1334 | 1.23 | 0.94 | 1.62 |
| **Stroke (yes)** | 0.0235 | 2.20 | 1.12 | 4.43 |
| **Hypertension (yes)** | 0.0014 | 1.48 | 1.16 | 1.88 |
| **Diabetes mellitus (yes)** | 0.0024 | 1.65 | 1.19 | 2.28 |
| **Coronary disease (yes)** | 0.0091 | 1.56 | 1.12 | 2.19 |
| **Hyperlipoidemia (yes)** | 0.1758 | 1.52 | 0.83 | 2.79 |
| **Kidney diseases (yes)** | 0.1877 | 1.53 | 0.81 | 2.90 |
| **Cancer (yes)** | 0.6511 | 1.12 | 0.68 | 1.82 |
| **Anemia (yes)** | 0.0273 | 1.71 | 1.06 | 2.77 |
| **Thyroid diseases (yes)** | 0.0001 | 2.07 | 1.44 | 2.99 |
| **Anti-hypertension drug (yes)** | 0.0310 | 1.34 | 1.03 | 1.74 |
| **Anti- diabetes drug (yes)** | 0.0122 | 1.60 | 1.11 | 2.33 |
| **Vitamins (yes)** | 0.0103 | 1.60 | 1.12 | 2.29 |
| **HAMA score (MAS)** | <0.0001 | 2.38 | 1.61 | 3.56 |
| **HAMD score (MDS)** | 0.0007 | 1.99 | 1.34 | 2.98 |
| **Sleep quality (bad)** | 0.0006 | 1.90 | 1.32 | 2.76 |
| **Sleep latency (minutes)** | 0.0038 | 1.01 | 1.00 | 1.02 |
| **Sleep duration (ref:≤5 hours)** | | |  |  |
| **5-6 hours** | 0.8805 | 0.96 | 0.59 | 1.58 |
| **6-7 hours** | 0.2105 | 0.74 | 0.47 | 1.18 |
| **7-8 hours** | 0.0141 | 0.55 | 0.34 | 0.89 |
| **>8 hours** | 0.0463 | 0.52 | 0.27 | 0.98 |
| **Bedtime (ref: Before 8:00 p.m)** | | |  |  |
| **8:00-9:00 p.m** | 0.8356 | 0.94 | 0.52 | 1.72 |
| **9:00 -10:00 p.m** | 0.4616 | 1.23 | 0.71 | 2.14 |
| **10:00-11:00 p.m** | 0.2566 | 1.41 | 0.78 | 2.56 |
| **After 11:00 p.m** | 0.0440 | 2.38 | 1.04 | 5.66 |
| **Sleep efficiency (≤70%)** | 0.2395 | 1.27 | 0.85 | 1.90 |
| **Sleep disorders** | 0.0379 | 1.46 | 1.02 | 2.10 |
| **Sleep assistance** | 0.0040 | 2.30 | 1.32 | 4.13 |
| **Day time dysfunction** | 0.0059 | 2.46 | 1.32 | 4.78 |
| **FBG (mmol/L)** | 0.3655 | 1.06 | 0.94 | 1.20 |
| **BUN (mmol/L)** | 0.1054 | 1.08 | 0.98 | 1.19 |
| **CR (μmol/L)** | 0.3717 | 1.00 | 0.99 | 1.01 |
| **UA (μmol/L)** | 0.9860 | 1.00 | 1.00 | 1.00 |
| **TG (mmol/L)** | 0.1400 | 0.90 | 0.79 | 1.03 |
| **TC (mmol/L)** | 0.0899 | 1.13 | 0.98 | 1.29 |
| **HDL-C (mmol/L)** | 0.6640 | 1.12 | 0.68 | 1.84 |
| **LDL-C (mmol/L)** | 0.0972 | 1.18 | 0.97 | 1.44 |

**Abbreviations:** OR, odds ratio; LCI, lower confidence interval (2.5%); UCI, upper confidence interval (97.5%); APOE *ε*4, apolipoprotein E ε4; FBG, Fasting blood glucose; BUN, blood urea nitrogen; CR, creatinine; UA, uric acid; TG, triglyceride; TC, total cholesterol; LDL-C, low density lipoprotein cholesterol; HDL-C, high density lipoprotein cholesterol; HAMA, Hamilton anxiety scale; MAS, Minimal anxiety symptoms; HAMD, Hamilton depression scale; MDS, Minimal depression symptoms; PSQI, Pittsburgh sleep quality index.

**Table S2. Multivariate logistic regression analysis for risk factors (Model 2)**

| **Factors** | **p** | **OR** | **LCI** | **UCI** |
| --- | --- | --- | --- | --- |
| **Age (≥65 years)** | <0.0001 | 1.74 | 1.33 | 2.27 |
| **Sex (female)** | 0.0449 | 1.30 | 1.01 | 1.69 |
| **Living alone (yes)** | 0.0032 | 2.29 | 1.33 | 4.04 |
| **Lack physical exercises (yes)** | 0.0065 | 1.42 | 1.10 | 1.82 |
| **Stroke (yes)** | 0.3095 | 1.46 | 0.71 | 3.08 |
| **Hypertension (yes)** | 0.0879 | 1.40 | 0.95 | 2.05 |
| **Diabetes mellitus (yes)** | 0.3870 | 1.32 | 0.70 | 2.47 |
| **Coronary disease (yes)** | 0.8489 | 1.04 | 0.71 | 1.51 |
| **Anemia (yes)** | 0.2019 | 1.39 | 0.84 | 2.32 |
| **Thyroid diseases (yes)** | 0.0022 | 1.82 | 1.24 | 2.69 |
| **Anti-hypertension drug (yes)** | 0.3130 | 0.81 | 0.53 | 1.23 |
| **Anti- diabetes drug (yes)** | 0.8665 | 0.94 | 0.47 | 1.90 |
| **Vitamins (yes)** | 0.0565 | 1.56 | 0.98 | 2.16 |
| **HAMA score (MAS)** | 0.0196 | 1.83 | 1.10 | 3.06 |
| **HAMD score (MDS)** | 0.4659 | 1.21 | 0.72 | 2.03 |
| **Sleep quality (bad)** | 0.1460 | 1.40 | 0.89 | 2.22 |
| **Sleep latency (minutes)** | 0.3422 | 1.00 | 0.99 | 1.01 |
| **Sleep duration (ref:≤5 hours)** | | |  |  |
| **5-6 hours** | 0.3730 | 1.28 | 0.74 | 2.21 |
| **6-7 hours** | 0.6278 | 1.14 | 0.67 | 1.97 |
| **7-8 hours** | 0.8894 | 0.96 | 0.55 | 1.69 |
| **>8 hours** | 0.7963 | 1.10 | 0.52 | 2.32 |
| **Bedtime (ref: Before 8:00 p.m)** | | |  |  |
| **8:00-9:00 p.m** | 0.9858 | 1.01 | 0.53 | 1.91 |
| **9:00 -10:00 p.m** | 0.5189 | 1.22 | 0.67 | 2.22 |
| **10:00-11:00 p.m** | 0.1429 | 1.64 | 0.85 | 3.18 |
| **After 11:00 p.m** | 0.0901 | 2.23 | 0.89 | 5.73 |
| **Sleep disorders** | 0.6304 | 1.10 | 0.74 | 1.66 |
| **Sleep assistance** | 0.4945 | 1.25 | 0.66 | 2.43 |
| **Day time dysfunction** | 0.0172 | 2.29 | 1.17 | 4.63 |
| **TC (mmol/L)** | 0.6538 | 1.11 | 0.71 | 1.72 |
| **LDL-C (mmol/L)** | 0.8852 | 1.05 | 0.56 | 1.97 |

**Abbreviations:** OR, odds ratio; LCI, lower confidence interval (2.5%); UCI, upper confidence interval (97.5%); TC, total cholesterol; LDL-C, low density lipoprotein cholesterol; HAMA, Hamilton anxiety scale; MAS, Minimal anxiety symptoms; HAMD, Hamilton depression scale; MDS, Minimal depression symptoms.

**Table S3. Multivariate linear regression analysis for risk factors (Model 3)**

| **Factors** | **p** | **Beta** | **LCI** | **UCI** |
| --- | --- | --- | --- | --- |
| **Age (≥65 years)** | 0.0091 | 0.07 | 0.02 | 0.12 |
| **Sex (female)** | 0.9816 | <0.01 | -0.05 | 0.05 |
| **Living alone (yes)** | 0.0921 | 0.09 | -0.01 | 0.19 |
| **Lack physical exercises (yes)** | 0.2759 | 0.03 | -0.02 | 0.07 |
| **Stroke (yes)** | 0.3097 | 0.07 | -0.06 | 0.19 |
| **Hypertension (yes)** | 0.1639 | 0.05 | -0.02 | 0.12 |
| **Diabetes mellitus (yes)** | 0.1332 | 0.09 | -0.03 | 0.21 |
| **Coronary disease (yes)** | 0.3176 | -0.04 | -0.11 | 0.03 |
| **Anemia (yes)** | 0.0262 | 0.10 | 0.01 | 0.19 |
| **Thyroid diseases (yes)** | 0.0004 | 0.13 | 0.06 | 0.20 |
| **Anti-hypertension drug (yes)** | 0.7140 | -0.01 | -0.09 | 0.06 |
| **Anti- diabetes drug (yes)** | 0.6942 | -0.03 | -0.16 | 0.11 |
| **Vitamins (yes)** | 0.0520 | 0.07 | 0.00 | 0.14 |
| **HAMA score (MAS)** | 0.0011 | 0.14 | 0.06 | 0.23 |
| **HAMD score (MDS)** | 0.5427 | 0.03 | -0.06 | 0.11 |
| **Sleep quality (bad)** | 0.1259 | 0.06 | -0.02 | 0.14 |
| **Sleep latency (minutes)** | 0.0565 | <0.01 | 0.00 | 0.00 |
| **Sleep duration (ref:≤5 hours)** |  |  |  |  |
| **5 -6 hours** | 0.9845 | <-0.01 | -0.09 | 0.09 |
| **6 -7 hours** | 0.3948 | 0.04 | -0.05 | 0.13 |
| **7- 8 hours** | 0.3943 | 0.04 | -0.05 | 0.14 |
| **>8 hours** | 0.8245 | 0.01 | -0.11 | 0.14 |
| **Bedtime (ref: Before 8:00 p.m)** |  |  |  |  |
| **8:00-9:00 p.m** | 0.4765 | -0.04 | -0.15 | 0.07 |
| **9:00 -10:00 p.m** | 0.8939 | 0.01 | -0.10 | 0.11 |
| **10:00-11:00 p.m** | 0.3816 | 0.05 | -0.06 | 0.16 |
| **After 11:00 p.m** | 0.4260 | 0.07 | -0.10 | 0.23 |
| **Sleep disorders** | 0.7898 | 0.01 | -0.06 | 0.08 |
| **Sleep assistance** | 0.6455 | 0.02 | -0.08 | 0.13 |
| **Day time dysfunction** | 0.0002 | 0.21 | 0.10 | 0.32 |
| **TC (mmol/L)** | 0.6994 | 0.02 | -0.07 | 0.10 |
| **LDL-C (mmol/L)** | 0.7297 | -0.02 | -0.14 | 0.10 |

**Abbreviations:** LCI, lower confidence interval (2.5%); UCI, upper confidence interval (97.5%); TC, total cholesterol; LDL-C, low density lipoprotein cholesterol; HAMA, Hamilton anxiety scale; MAS, Minimal anxiety symptoms; HAMD, Hamilton depression scale; MDS, Minimal depression symptoms.

**Table S4. Subgroup analyses stratified by sex**

| **Factors** | **Male** | | | | **Female** | | | |
| --- | --- | --- | --- | --- | --- | --- | --- | --- |
|  | **p** | **OR** | **LCI** | **UCI** | **p** | **OR** | **LCI** | **UCI** |
| **Age(≥65 years)** | 0.0004 | 1.90 | 1.33 | 2.70 | 0.0451 | 1.54 | 1.01 | 2.35 |
| **Living alone (yes)** | 0.1207 | 1.75 | 0.86 | 3.56 | 0.0060 | 4.13 | 1.61 | 12.73 |
| **Lack physical exercises (yes)** | 0.0315 | 1.44 | 1.03 | 2.01 | 0.0810 | 1.42 | 0.96 | 2.12 |
| **Stroke (yes)** | 0.1989 | 1.92 | 0.72 | 5.49 | 0.9708 | 0.98 | 0.33 | 3.04 |
| **Hypertension (yes)** | 0.2401 | 1.37 | 0.81 | 2.30 | 0.2429 | 1.40 | 0.79 | 2.49 |
| **Diabetes mellitus (yes)** | 0.3957 | 1.40 | 0.64 | 3.03 | 0.7613 | 1.18 | 0.39 | 3.64 |
| **Coronary disease (yes)** | 0.8841 | 1.04 | 0.62 | 1.74 | 0.9365 | 0.98 | 0.55 | 1.73 |
| **Anemia (yes)** | 0.3776 | 1.50 | 0.60 | 3.77 | 0.2327 | 1.46 | 0.78 | 2.76 |
| **Thyroid diseases (yes)** | 0.0646 | 1.71 | 0.97 | 3.04 | 0.0116 | 1.99 | 1.17 | 3.42 |
| **Anti-hypertension drug (yes)** | 0.4655 | 0.81 | 0.45 | 1.44 | 0.4769 | 0.80 | 0.43 | 1.49 |
| **Anti- diabetes drug (yes)** | 0.6305 | 0.81 | 0.34 | 1.93 | 0.7731 | 1.19 | 0.35 | 4.01 |
| **Vitamins (yes)** | 0.1313 | 1.48 | 0.89 | 2.48 | 0.2705 | 1.39 | 0.77 | 2.51 |
| **HAMA score (MAS)** | 0.0067 | 2.83 | 1.35 | 6.11 | 0.6180 | 1.20 | 0.58 | 2.49 |
| **HAMD score (MDS)** | 0.9050 | 1.05 | 0.49 | 2.20 | 0.3576 | 1.41 | 0.68 | 2.98 |
| **Sleep quality (bad)** | 0.2636 | 1.41 | 0.77 | 2.57 | 0.3921 | 1.40 | 0.65 | 3.02 |
| **Sleep latency (minutes)** | 0.1118 | 1.01 | 0.99 | 1.02 | 0.9721 | 1.00 | 0.99 | 1.01 |
| **Sleep duration (ref:≤5 hours)** |  |  |  |  |  |  |  |  |
| **5-6 hours** | 0.6992 | 1.17 | 0.53 | 2.60 | 0.5413 | 1.28 | 0.58 | 2.85 |
| **6-7 hours** | 0.6675 | 1.18 | 0.55 | 2.57 | 0.9483 | 1.03 | 0.46 | 2.29 |
| **7-8 hours** | 0.8245 | 0.91 | 0.42 | 2.02 | 0.9297 | 0.96 | 0.42 | 2.24 |
| **>8 hours** | 0.9597 | 0.97 | 0.35 | 2.69 | 0.6303 | 1.33 | 0.42 | 4.20 |
| **Bedtime (ref: Before 8:00 p.m)** | | |  |  |  |  |  |  |
| **8:00-9:00 p.m** | 0.9890 | 1.01 | 0.42 | 2.47 | 0.8466 | 0.91 | 0.34 | 2.45 |
| **9:00 -10:00 p.m** | 0.5409 | 1.29 | 0.57 | 2.98 | 0.8549 | 1.09 | 0.43 | 2.77 |
| **10:00-11:00 p.m** | 0.1941 | 1.83 | 0.74 | 4.66 | 0.7541 | 1.18 | 0.42 | 3.32 |
| **After 11:00 p.m** | 0.2158 | 2.18 | 0.64 | 7.62 | 0.1821 | 2.77 | 0.65 | 13.46 |
| **Sleep disorders** | 0.7843 | 1.08 | 0.63 | 1.85 | 0.7110 | 1.13 | 0.60 | 2.14 |
| **Sleep assistance** | 0.1203 | 2.33 | 0.83 | 7.23 | 0.6723 | 0.83 | 0.34 | 2.01 |
| **Day time dysfunction** | 0.2496 | 1.68 | 0.70 | 4.17 | 0.0389 | 3.23 | 1.12 | 10.80 |
| **TC (mmol/L)** | 0.7471 | 0.90 | 0.48 | 1.67 | 0.3846 | 1.33 | 0.70 | 2.55 |
| **LDL-C (mmol/L)** | 0.5813 | 1.28 | 0.54 | 3.15 | 0.8039 | 0.89 | 0.35 | 2.23 |

**Abbreviations:** OR, odds ratio; LCI, lower confidence interval (2.5%); UCI, upper confidence interval (97.5%); TC, total cholesterol; LDL-C, low density lipoprotein cholesterol; HAMA, Hamilton anxiety scale; MAS, Minimal anxiety symptoms; HAMD, Hamilton depression scale; MDS, Minimal depression symptoms.

**Table S5. Subgroup analyses stratified by age**

| **Factors** | **Mid-life** | | | | **Late-life** | | | |
| --- | --- | --- | --- | --- | --- | --- | --- | --- |
|  | **p** | **OR** | **LCI** | **UCI** | **p** | **OR** | **LCI** | **UCI** |
| **Sex (female)** | 0.1247 | 1.31 | 0.93 | 1.85 | 0.2626 | 1.25 | 0.84 | 1.87 |
| **Living alone (yes)** | 0.0890 | 2.09 | 0.89 | 4.98 | 0.0153 | 2.50 | 1.22 | 5.43 |
| **Lack physical exercises (yes)** | 0.0751 | 1.36 | 0.97 | 1.92 | 0.0369 | 1.50 | 1.03 | 2.20 |
| **Stroke (yes)** | 0.1419 | 3.05 | 0.72 | 15.75 | 0.7058 | 1.18 | 0.51 | 2.79 |
| **Hypertension (yes)** | 0.0517 | 1.68 | 0.99 | 2.81 | 0.6396 | 1.15 | 0.65 | 2.04 |
| **Diabetes mellitus (yes)** | 0.1987 | 1.87 | 0.72 | 4.97 | 0.9580 | 1.02 | 0.45 | 2.34 |
| **Coronary disease (yes)** | 0.7039 | 0.87 | 0.42 | 1.74 | 0.6264 | 1.12 | 0.71 | 1.78 |
| **Anemia (yes)** | 0.1892 | 1.55 | 0.80 | 3.00 | 0.6912 | 1.18 | 0.53 | 2.66 |
| **Thyroid diseases (yes)** | 0.0051 | 2.05 | 1.24 | 3.41 | 0.1441 | 1.56 | 0.87 | 2.86 |
| **Anti-hypertension drug (yes)** | 0.1911 | 0.67 | 0.36 | 1.22 | 0.8886 | 0.96 | 0.53 | 1.72 |
| **Anti- diabetes drug (yes)** | 0.5334 | 0.70 | 0.22 | 2.15 | 0.7947 | 1.13 | 0.45 | 2.79 |
| **Vitamins (yes)** | 0.0856 | 1.54 | 0.94 | 2.53 | 0.2858 | 1.39 | 0.76 | 2.57 |
| **HAMA score (MAS)** | 0.1854 | 1.60 | 0.80 | 3.20 | 0.0348 | 2.41 | 1.08 | 5.62 |
| **HAMD score (MDS)** | 0.3460 | 1.41 | 0.68 | 2.89 | 0.7798 | 1.12 | 0.49 | 2.56 |
| **Sleep quality (bad)** | 0.6354 | 1.17 | 0.60 | 2.26 | 0.1695 | 1.60 | 0.82 | 3.17 |
| **Sleep latency (minutes)** | 0.3165 | 1.00 | 0.99 | 1.01 | 0.7110 | 1.00 | 0.99 | 1.01 |
| **Sleep duration (ref:≤5 hours)** |  |  |  |  |  |  |  |  |
| **5-6 hours** | 0.4497 | 1.34 | 0.63 | 2.87 | 0.8361 | 1.09 | 0.47 | 2.55 |
| **6-7 hours** | 0.8237 | 1.09 | 0.51 | 2.34 | 0.9834 | 1.01 | 0.45 | 2.29 |
| **7-8 hours** | 0.6579 | 0.83 | 0.37 | 1.89 | 0.9913 | 1.00 | 0.44 | 2.30 |
| **>8 hours** | 0.5569 | 1.36 | 0.48 | 3.81 | 0.5895 | 0.74 | 0.24 | 2.23 |
| **Bedtime (ref: Before 8:00 p.m)** | | |  |  |  |  |  |  |
| **8:00-9:00 p.m** | 0.2125 | 1.92 | 0.71 | 5.56 | 0.2667 | 0.62 | 0.26 | 1.44 |
| **9:00 -10:00 p.m** | 0.3985 | 1.52 | 0.59 | 4.15 | 0.9031 | 1.05 | 0.47 | 2.37 |
| **10:00-11:00 p.m** | 0.3185 | 1.67 | 0.62 | 4.74 | 0.1179 | 2.22 | 0.82 | 6.14 |
| **After 11:00 p.m** | 0.0616 | 3.36 | 0.96 | 12.46 | 0.6548 | 1.42 | 0.31 | 6.97 |
| **Sleep disorders** | 0.7902 | 1.08 | 0.61 | 1.92 | 0.8393 | 1.06 | 0.58 | 1.94 |
| **Sleep assistance** | 0.2073 | 2.08 | 0.68 | 6.88 | 0.9037 | 0.95 | 0.42 | 2.20 |
| **Day time dysfunction** | 0.0014 | 3.92 | 1.74 | 9.41 | 0.7336 | 0.81 | 0.23 | 2.85 |
| **TC (mmol/L)** | 0.3090 | 1.33 | 0.76 | 2.33 | 0.5580 | 0.81 | 0.38 | 1.66 |
| **LDL-C (mmol/L)** | 0.8088 | 0.91 | 0.42 | 2.01 | 0.5537 | 1.36 | 0.49 | 3.93 |

**Abbreviations:** LCI, lower confidence interval (2.5%); UCI, upper confidence interval (97.5%); TC, total cholesterol; LDL-C, low density lipoprotein cholesterol; HAMA, Hamilton anxiety scale; MAS, Minimal anxiety symptoms; HAMD, Hamilton depression scale; MDS, Minimal depression symptoms.

**Figure S1. Risk factors for SCD-plus**

**
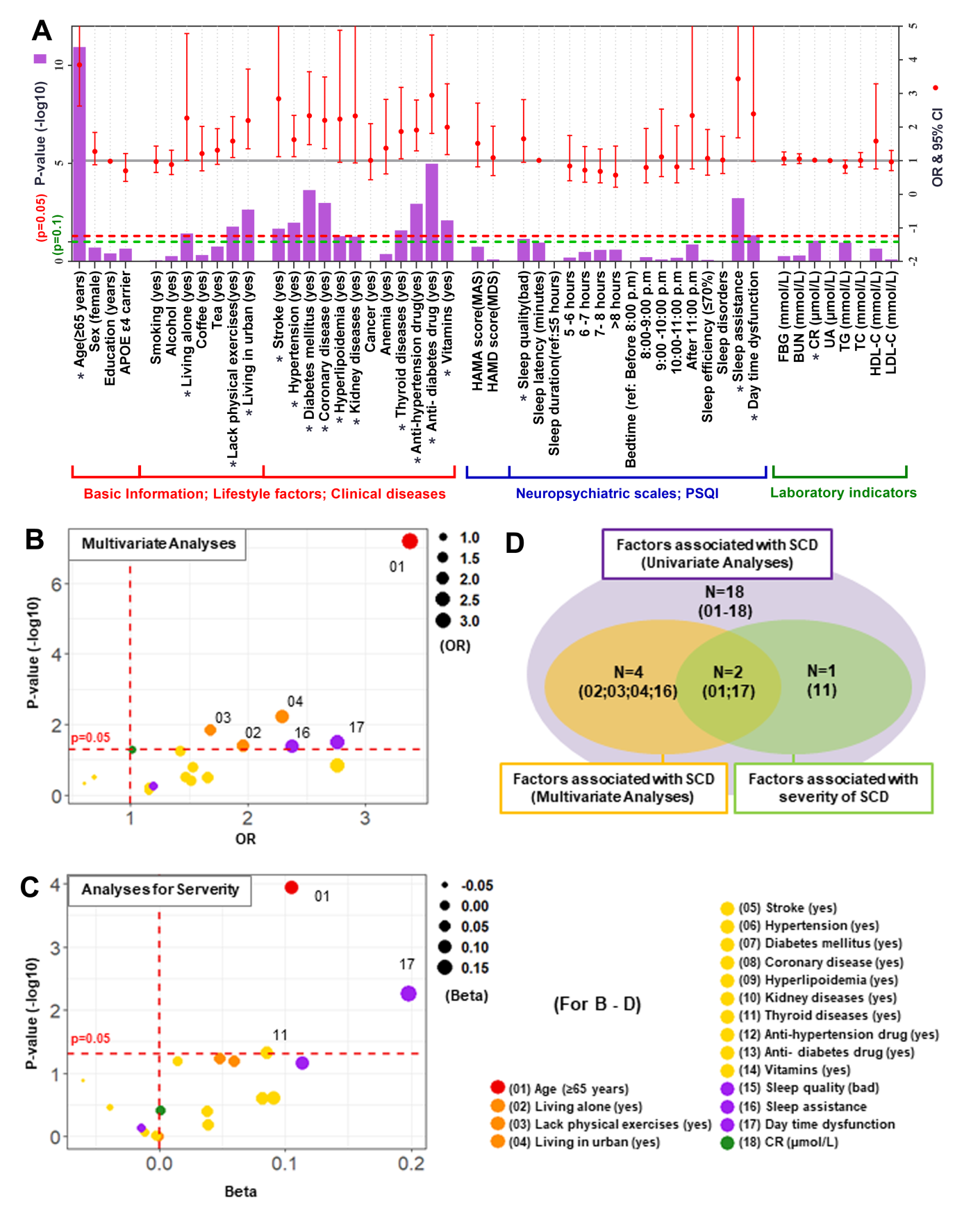
**

**Abbreviations:** OR, odds ratio; LCI, lower confidence interval (2.5%); UCI, upper confidence interval (97.5%); APOE ε4, apolipoprotein E ε4; FBG, Fasting blood glucose; BUN, blood urea nitrogen; CR, creatinine; UA, uric acid; TG, triglyceride; TC, total cholesterol; LDL-C, low density lipoprotein cholesterol; HDL-C, high density lipoprotein cholesterol; HAMA, Hamilton anxiety scale; MAS, Minimal anxiety symptoms; HAMD, Hamilton depression scale; MDS, Minimal depression symptoms; PSQI, Pittsburgh sleep quality index.

Risk factors for SCD-plus were determined using three models. (A) Univariate logistic regression models (Model 1) were used to test association of each factor with the risk of SCD. (B) Then all the significant factors in univariate models (*p <0.1) were included in the multivariate logistic regression (Model 2) to test their associations with the SCD-plus status. (C) Similarly, all the significant factors in univariate models (*p <0.1) were also included in the multivariate linear regression (Model 3) to test their associations with the SCD-plus severity. The age, sex, years of education and *APOE ε4* status were included in two multivariate models as the basic covariates, regardless of their results in Model 1. (D) The results of three models were summarized in a Venn diagram.
